# Supplementary material for: Short-Term Dosage Regimen for Stimulation-Induced Long-Lasting Desynchronization
Source: Front Physiol. 2018 Apr 12;9:376. doi: 10.3389/fphys.2018.00376 (PMC5906576; doi:10.3389/fphys.2018.00376)
Supplement: Supplementary file 1 [file DataSheet1.DOCX]

Supplementary Material

Short-term dosage regimen for stimulation-induced long-lasting desynchronization

Thanos Manos*, Magteld Zeitler, Peter A. Tass

*** Correspondence:** Thanos Manos: t.manos@fz-juelich.de


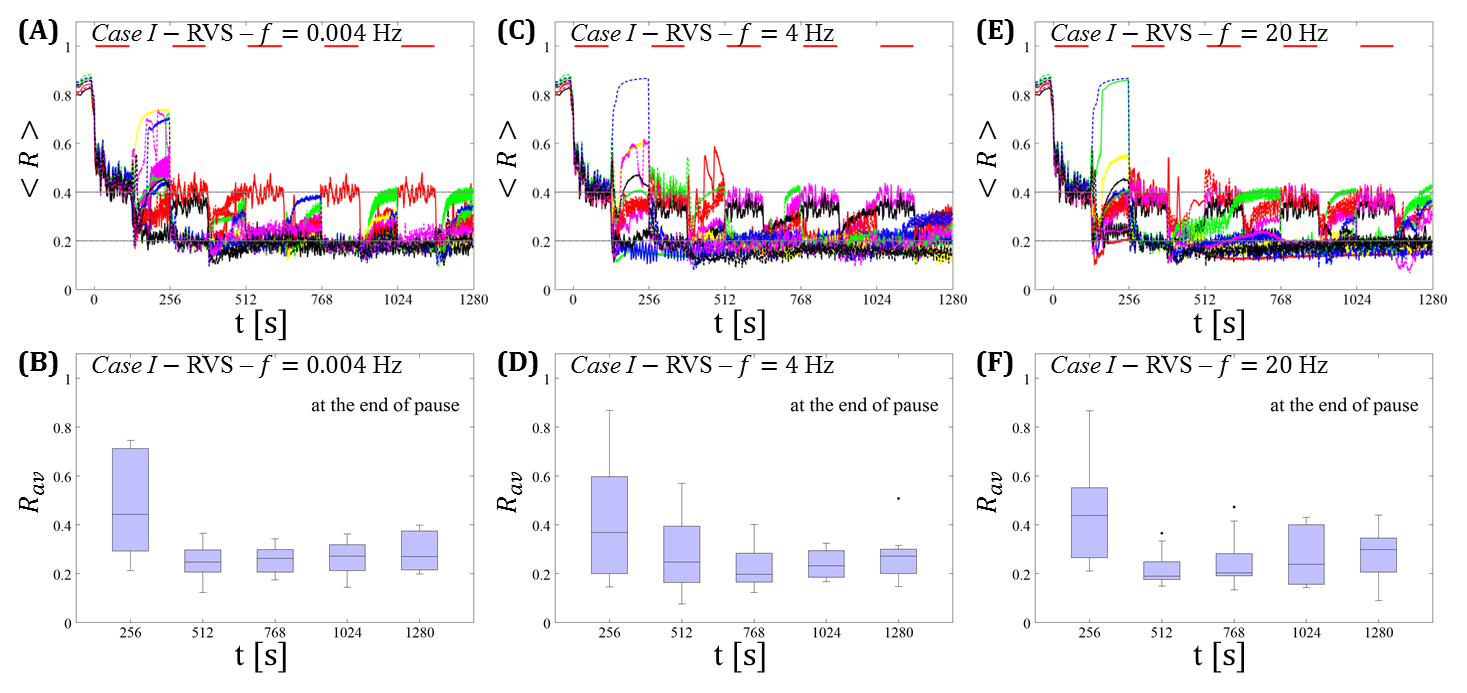


**Supplementary Figure 1.** Protocol C in the presence of intrinsic variations of the firing rates caused by a modulatory low-amplitude current input $I_{var}=A\cdot sin(2\pi\cdot f\cdot t)$, with$A=1$, $f=0.004$ Hz **(A, B)**, $f=4$ Hz **(C, D)** and $f=20$ Hz **(E, F)***.* Spaced multishot RVS CR stimulation with demand-controlled random variation of the stimulation period $T_{s}$and with demand-controlled variation of the intensity. The low-amplitude variation $I_{\mathrm{var}}$ is active during the entire simulations, respectively. Its low amplitude $A=1$ ensures that the dynamics of the network is not drastically affected. **(A, C, E)** Time evolution of the order parameter $<R>$ averaged over a sliding window during 5 consecutive RVS CR shots respectively. If $R_{av}$ at the end of a pause exceeds 0.4, the CR stimulation period of the subsequent SVS shot is decreased by $T_{s}\to T_{s}-$1 ms (see text). **(B, D, F)** Boxplots for the time-averaged order parameter$R_{av}$ at the end of each pause, illustrate the overall outcome for all tested 11 networks respectively. The horizontal solid red lines indicate the CR shots, while the horizontal dashed grey lines highlight the two control thresholds (see text). *Case I* stimulation parameters are unfavourable for anti-kindling: $(K, T_{s})=(0.30,11)$ (see text). Format as in **Figure 9**.


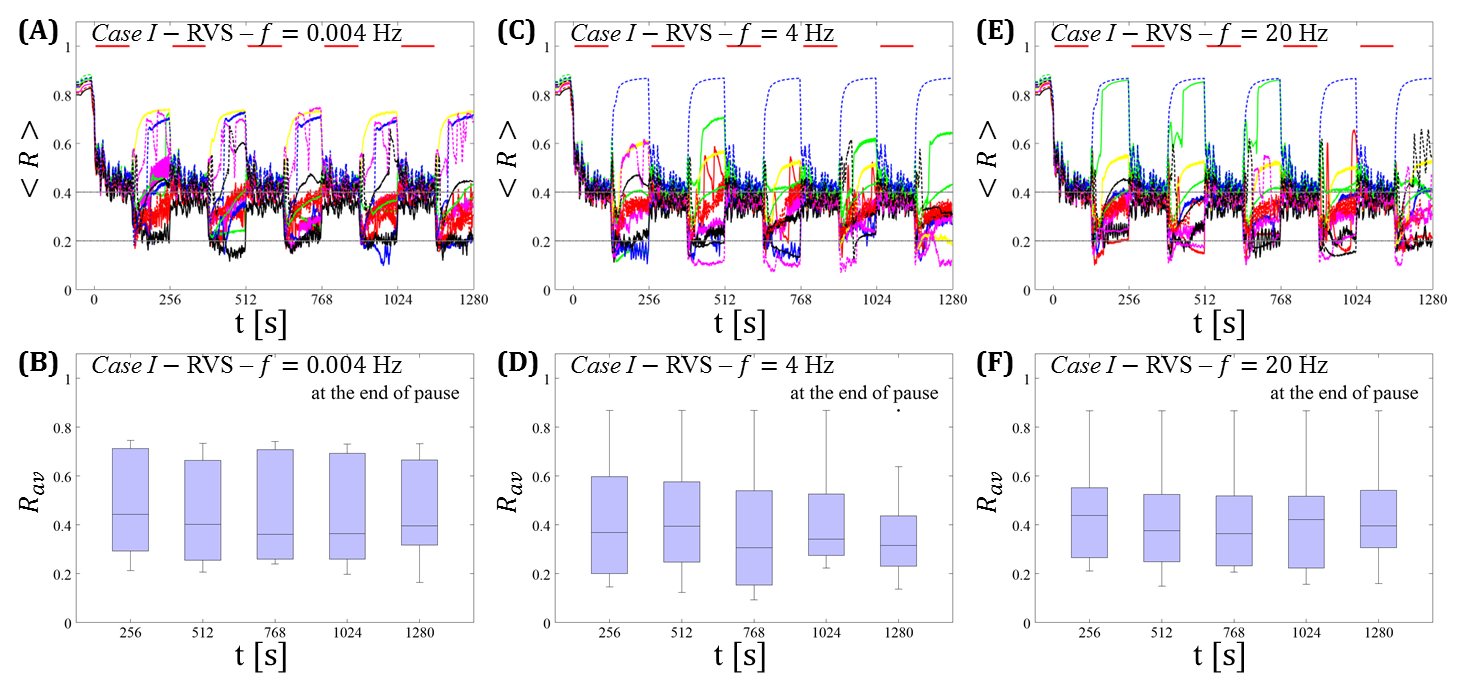


**Supplementary Figure 2.** Protocol A in the presence of intrinsic variations of the firing rates caused by a modulatory low-amplitude current input $I_{var}=A\cdot sin(2\pi\cdot f\cdot t)$, with$A=1$, $f=0.004$ Hz **(A, B)**, $f=4$ Hz **(C, D)** and $f=20$ Hz **(E, F)**. Spaced multishot RVS CR stimulation with fixed stimulation period $T_{s}$. Same series of simulations and analysis as in Supplementary Figure 1. **(A, C, E)** Time evolution of the order parameter $<R>$ averaged over a sliding window during 5 consecutive RVS CR shots respectively. **(B, D, F)** Boxplots for the time-averaged order parameter$R_{av}$at the end of each pause, illustrate the overall outcome for all tested 11 networks respectively. Spacing is symmetrical, i.e. CR shots and consecutive pauses are of the same duration. *Case I* stimulation parameters are unfavourable for anti-kindling: $(K, T_{s})=(0.30,11)$ (see text). Format as in **Figure 9**.

In our model, we have additionally compared our findings to the case of 8 stimulation sites and found no major differences. As an example, in Supplementary Figure 3 we show the time evolution of the order parameter $<R>$ averaged over a sliding window (panel A) and mean synaptic connectivity $C_{av}$ (panel C) for total CR-on time duration $t=128$ s using RVS CR stimulation signals and “optimal” parameters$\left( K,T_{s} \right)=\left( 0.20,10 \right)$ and 4 uniformly arranged on the network ring stimulation sites (see *Model and Network Description* section). Panels B and D show the time evolution of the order parameter $<R>$ averaged over a sliding window and mean synaptic connectivity $C_{av}$ for total CR-on time duration $t=128$ s using RVS CR stimulation signals for the same CR parameters for 8 uniformly arranged on the network ring stimulation sites respectively. The overall outcome is not improved. The mean synaptic connectivity of the neuronal population at time $t$ is calculated by the following equation (averaged over the neuron population):

$$C_{av}\left( t \right)=N^{-2}\sum_{i,j} sgn\left( M_{ij} \right)c_{ij}\left( t \right) ,$$

where $M_{ij}$ is defined in Equation 3 of our manuscript and sgn is the sign-function [see also (Popovych and Tass, 2012;Zeitler and Tass, 2015)].


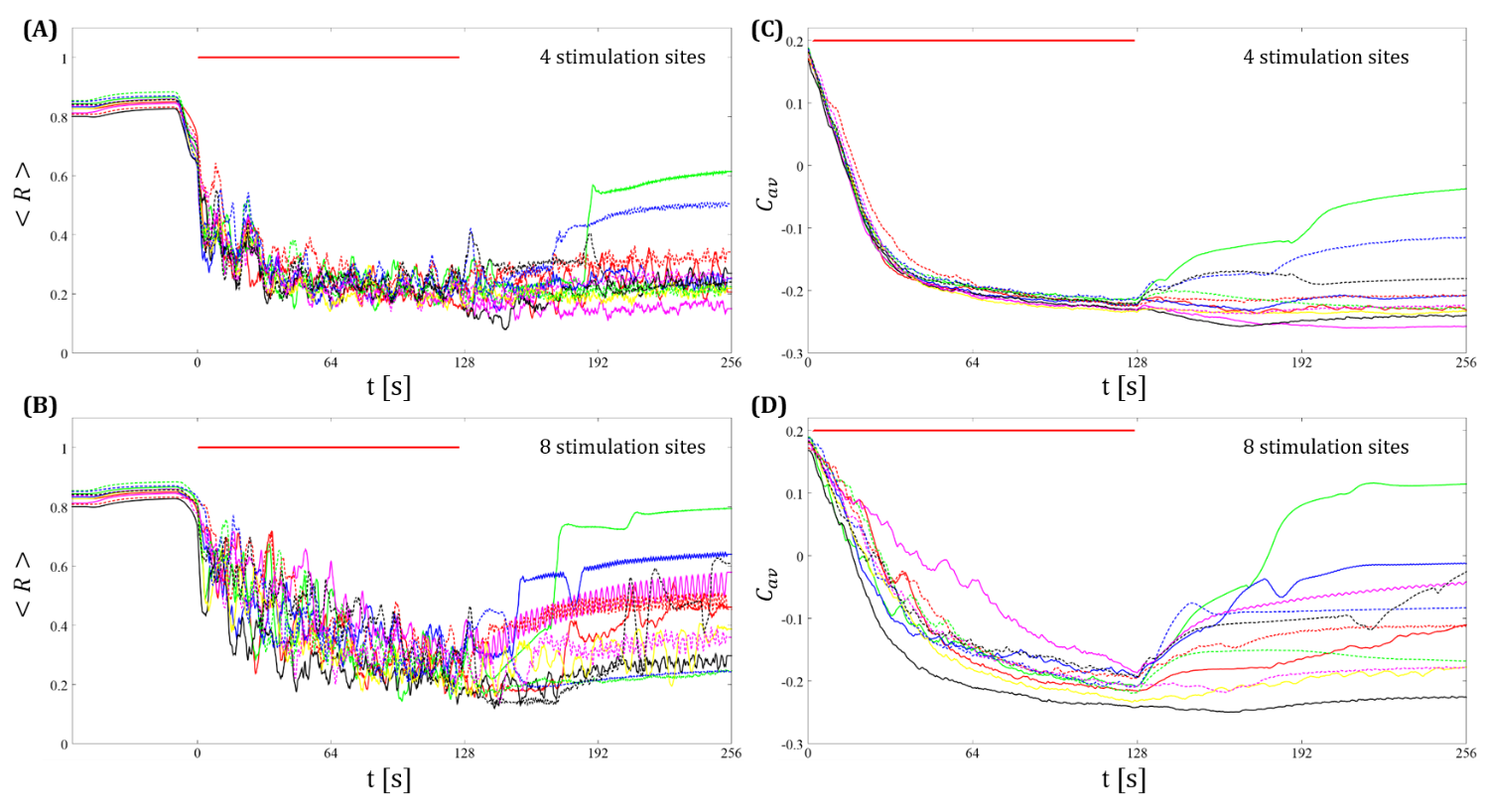


**Supplementary Figure 3.** Comparison using 4 vs 8 stimulation sites. **(A,C)** Time evolution of the order parameter $<R>$ averaged over a sliding window and mean synaptic connectivity $C_{av}$ for total CR-on time duration $t=128$ s using RVS CR stimulation signals and “optimal” parameters$\left( K,T_{s} \right)=\left( 0.20,10 \right)$ and 4 uniformly arranged on the network ring stimulation sites (see *Model and Network Description* section). **(B,D)** Time evolution of the order parameter $<R>$ averaged over a sliding window and mean synaptic connectivity $C_{av}$ for total CR-on time duration $t=128$ s using RVS CR stimulation signals for the same CR parameters for 8 uniformly arranged on the network ring stimulation sites.
